# Mr. Guthrie on the Anatomy and Diseases of the Neck of the Bladder, &c.

**Published:** 1835-01-01

**Authors:** 


### On the Anatomy and Diseases of the Neck of the Bladder and of the Urethra: being the Substance of the Lectures delivered in the Theatre of the Royal College of Surgeons in the Year 1830; and in the Westminster Hospital in 1833 and 1834. By *G. J. Guthrie*, F.R.S. Surgeon to the Westminster Hospital, and to the Royal Westminster Ophthalmic Hospital, &c. &c. &c. Octavo, pp. 284. London, 1834.

The diseases of the urinary organs have been investigated with much ardour and with some success by surgeons and physicians of the present day. Chemistry has done something and pathology has done more, to discriminate the affections of particular portions of the urinary apparatus, and to found on an accurate diagnosis a rational system of treatment.

If we look through the pages of the older writers, surgical and medical, we perceive the diseases of the kidney, the bladder, and urethra lumped together—symptoms erected into substantive complaints—and substantive complaints degraded into symptoms. Irritable bladder and catarrhus vesicæ are terms indiscriminately and generally employed, vague in their meaning, erroneous in their application, and pernicious in their influence.

Morbid anatomy has irrevocably banished those general assumptions and those idle notions, which were formed and fostered on a superficial examination of symptoms. Yet morbid anatomy has effected no more in this than in other departments of medicine. Physic and surgery have alike felt its power, and alike submitted to its revolutionary hand. There are some who look back with fondness and regret on the days of the Hippocratists and the barber-surgeons, who sigh at the downfall of that wholesome influence which dictated from the dogmatic chair of physic the handicraft of obedient surgery, and fettered infant science with the cobwebs of scholastic jargon and Aristotelian subtleties. The pedant writer may still exist, who would smother the lusty facts of Nature in the swaddling and the blankets of laborious art—a scriblerus who has lived upon the dusty shelf, and fed, moth-like, on the musty page—a critic who sneers folios and thinks black-letter —a biblical monstrosity, inopportunely dragged from the library and the museum to mingle with the beings of the open day.

Who would not laugh if such a man there be?

The works of Dr. Prout and of Sir Benjamin Brodie, so recent in their appearance and so valuable in their materials, would seem to render the chances of success on the part of other writers problematical, at least for the present. Yet so earnest is the competition in every branch of science and of art, that the triumph of one man induces others to attempt to share the laurels and divide the spoils. The knocks at the door of the temple of Fame are the more incessant the less the clamorous applicant is answered.

Mr. Guthrie informs his readers in an advertisement, that many years have elapsed since a deputation from the medical officers of the army requested he would publish the lectures he had delivered to them on the diseases of the urinary and sexual organs. The lapse of those years has given time for observation, and has been productive of experience. He therefore offers this, the first part of his work, more worthy than it then was of the attention of the public, and the approbation of his friends.

The work consists of sixteen lectures. They are partly composed of anatomical description, chiefly of surgical remark. The descriptive portion is confined to the urethra and the neck of the bladder; the surgical consists of observations upon stricture and the consequences that result from it, and on various diseases of the prostate gland. The large amount of descriptive anatomy will preclude our offering an analysis of these lectures. We shall endeavour to present a consistent account of their more important or more novel contents. The talents and the opportunities of Mr. Guthrie ensure an attentive consideration of his opinions.

The first lecture is almost exclusively devoted to the structure of the bladder. Mr. Guthrie's investigations appear to have been minute, and are probably correct. Much of what he states is to be found in the best anatomical works, especially in those of the modern French school; but some of the descriptions, the conclusions, and the speculations, would seem to be his own.

Mr. Guthrie presents a particular account of what the French have denominated the *trigone*, a triangular space included between the *luette*, or the verumontanum, in front, and a line which may be drawn from one orifice of the ureters to the other behind. This triangular part is composed of a peculiar elastic substance, which surrounds the orifices of the ureters. We mention these circumstances for the purpose of introducing Mr. Guthrie's opinions on the mode in which the urine is received into the bladder. He observes that the orifice of the ureter is less distensible than the remainder of that canal; and that it still remains of its usual size when the latter has become much enlarged by the continued pressure of urine retained in it. The passage of calculi into the bladder is frequently obstructed at the orifice of the ureter.

"If the stone is large it sometimes sticks in the orifice and cannot pass through, giving rise to continued inconvenience, and to the symptoms of stone in the bladder from sympathy. This is exemplified by a preparation in the museum of the Royal College of Surgeons. The patient, a gentleman, consulted several surgeons of eminence, was sounded, the stone was detected, the operation was declared to be necessary, but was deferred for two or three weeks, until he should improve a little in health. This however he did not do, but on the contrary gradually got worse and died. On examination the stone was found sticking in the orifice of the left ureter, as I have described it. If an operation had been done, the stone would probably not have been extracted, its situation might not even have been detected; and it shows the propriety and necessity of not only being able to feel a stone *distinctly*, but also to be able to move it *distinctly* with the sound before an operation is resorted to." 6.

Mr. Guthrie alludes to those linear bands which descend from the ureter on either side and pass into the prostate, or are attached behind the uvula. These, as our readers are probably aware, have been considered muscles by Sir Charles Bell, who thought that they were intended to protect the orifices from pressure and obliteration, and maintain them in a patulous condition. Mr. Guthrie seems to think that they are better fitted for keeping the trigone fixed and for strengthening and raising it up when necessary, than for acting on the ureters, which, being surrounded by an elastic substance, require no peculiar apparatus to preserve them open. Both speculations may safely be left to the judgment or the fancy of those who wish to solve the knotty problem.

The trigone is well known to be extremely sensitive, and the pain or irritation sometimes experienced on the entrance of an instrument within the bladder, has been thought to be owing to its contact with this part. But Mr. Guthrie offers some reasons for doubting, indeed for denying the justice of this opinion. The trigone is seated at the posterior and inferior portion of the bladder, and the point of an instrument, when the organ is moderately full, would only touch the very apex of the part in question. It is probable that the irritation is excited by the instrument touching the prostatic division of the urethra.

The trigone enjoys an important surgical relation. It is here that the trocar is introduced into the bladder, in the operation of puncturing the latter through the rectum. To this operation Mr. Guthrie urges the following objection.

"The operation of puncturing the bladder through the rectum was founded on the supposed anatomical fact, that the triangular space rested on, and closely adhered to the rectum, so that the urine would flow direct from one into the other, without escaping into the neighbouring structures; but it has been shown, that the peritoneum instead of passing down behind the bladder, and between it and the rectum to the base of the triangular space, and there terminating in a cul de sac, does sometimes pass on further, between the triangular space and the rectum, and even occasionally up to the prostate gland; so that by puncturing through the rectum the general cavity of the peritoneum is opened before the bladder is penetrated, and the patient in such case must be lost, from the urine finding its way into the peritoneal cavity, and giving rise to an inflammation which has always been destructive whenever this kind of effusion has taken place, and from whatever cause. I am not aware of there being any signs by which this conformation can or cannot be distinguished, and consequently, if that opinion be correct, the safety of the patient depends not on the knowledge and ability of the surgeon, but on an accidental although the more usual formation of the part on which the operation is performed." 11.

This objection is highly deserving of attention, yet we are not aware that many accidents of the nature alluded to by Mr. Guthrie have followed the performance of the operation through the rectum.

Mr. Guthrie proceeds to describe with minuteness, and, we suppose, with accuracy, the longitudinal muscular fibres of the bladder. Yet, perhaps, the description contains little of general interest, and is chiefly adapted for the attention or the criticism of those engaged in anatomical pursuits. The question of the existence of a sphincter, is more definite and more important. Sir Charles Bell conceived that he could demonstrate it, but Mr. Guthrie has failed to discover it. He is of opinion that the portion of the bladder surrounding the opening into the urethra possesses but little muscular contractility, whilst it is endowed with a considerable degree of elasticity, which any one may ascertain by stretching the part. When the two muscular layers of the bladder contract, its tendons inserted into the pubes, become with the prostate generally the fixed points, the urine is forced against the orifice of the urethra, which yields by its elasticity, and returns to its former state when the pressure is removed. But circumstances appear to shake Mr. Guthrie's firmness, and he grants that it is possible that the neck of the bladder may be muscular *and* elastic.

Mr. Guthrie understands by " neck of the bladder," the small part surrounding the very opening itself into the urethra—a ring a little broader or thicker than the bladder itself—that portion on which the uvula is situated, the urethra being before and the bladder behind it. It is necessary to state, and to remember this, for, in common anatomical parlance, the neck of the bladder signifies that portion included in the prostate.

With a notice of one other point, we conclude the first lecture of the work. The point in question is this :—that in cases in which the urine is suppressed, apoplexy, or rather coma, is the consequence. Mr. Guthrie remarks, that all persons in whom the suppression is complete, die paralytic and apoplectic. He presents an illustration of the fact, in the instance of a lady affected with cancer of the uterus. The disease, after a time, extended towards the ureters, which at last were embraced and pressed upon by it as they entered the bladder. The lady, as this took place, began to suffer more than commonly from derangement in her urinary apparatus; the bladder was found ultimately, on passing the catheter, to contain little or no water; she fell into a state of low fever, became paralytic, afterwards comatose, and died paralytic and apoplectic. On examination, the ureters were found impervious at the part where they were grasped by the diseased structure ; above this they were greatly enlarged, the kidneys were also dilated.

It appears to us, that Mr. Guthrie's expression might advantageously be rendered more precise. Patients in whom the urine is suppressed, retained, or extravasated into textures not intended for its reception, evince a remarkable, if not a constant, disposition to coma and to serous effusion in the brain. It has long been known that suppression of the secretion gives rise to symptoms of this description ; but it is not, we believe, so generally understood, that other affections of the urinary apparatus not unfrequently occasion the same effect. Retention of urine may be readily supposed to produce sequelæ very similar to those resulting from suppression, for retention must itself give rise to suppression. But it might not, à priori, appear so probable that chronic inflammation and abscess of the kidney—or urinary extravasation into the cellular tissue—or that chronic abscess in the pelvic fascia, following lithotomy, or connected with stricture would severally be attended, in their latter stages, with symptoms of cerebral effusion. Yet such is the fact, and, leaving the explanation for the present to others, we content ourselves with barely stating it here.

The second lecture is devoted to the anatomy of the prostate, to a glance at the diseases of the elastic structure of the neck of the bladder, and to the symptoms of the pouches occasionally formed in the parietes of the latter.

The anatomy of the prostate may be left to the anatomists. Mr. Guthrie disproves, what has been already universally disallowed, the claim of Sir E. Home to be deemed the discoverer of the third lobe of the gland in question. Mr. Guthrie mentions some curious circumstances connected with the preparations of the prostate, now in the Hunterian Museum of the College. But we pass to some practical deductions drawn by Mr. Guthrie, from the anatomical data which he offers. Those deductions are as follow.

1. That the elastic structure at the neck of the bladder may be diseased without any necessary connexion with the prostate gland.

2. That the prostate may be diseased without any necessary connexion with the elastic structure.

In proof of the preceding statements, Mr. Guthrie exhibits to his pupils the preparation, and to the profession a drawing, of disease of the elastic structure of the bladder, independent of any affection of the prostate. He also alludes to another case, in which the circumstances were just reversed, the prostate being enlarged and the elastic structure unaffected. The right lobe of the gland was the part most augmented in dimensions, nearly closing the vesical orifice into the urethra, pushing the latter to the left, and drawing up the mucous membrane of the bladder, so as to form a bar across its under part. Had the bulk of the prostate been reduced, or the bar of mucous membrane been removed, the obstruction to the flow of urine would, in either case, have been diminished.

When a third lobe of the prostate projects into the bladder, the elastic structure is usually implicated, and constitutes a hard, firm bank, in addition to the nipple-like valve of the former. Between these, the retention of urine may become complete, and the complication is much less curable than disease of the neck of the bladder alone.

"In its simple or first stage, when there is only a defect of elasticity, it gives rise to stricture at the very neck or orifice of the bladder, curable by common means, if properly applied. In its second stage, when the bar is formed and becomes more or less rigid, a small bougie rests against it, and if made of soft materials bends, and cannot be made to proceed; if a solid instrument, it passes in one of the hollows on each side of the white central line, which are also deepened by the elevation of the uvula vesicæ, catches on the valve at the entrance, and when the handle of the instrument is depressed, it raises it, bladder, rectum and all, upon its point, until the pain or the resistance induces the surgeon to forego the depression, or the valve yields or is torn, when it finds its way into the bladder; or perhaps the surgeon, not possessing much experience, is satisfied with the distance the strument has gone in, and supposes he has passed it into the bladder. This is one of the evils which arises from the attention which has been paid to the length of the urethra with regard to inches only; for when a man is told that the urethra is often only eight inches long, and finds his instrument has passed perhaps more than nine, he may deceive himself, although his patient is not relieved. I had a gentleman from America under my care lately under these circumstances. He had never passed his bougie beyond the neck of the bladder, although he and his surgeon supposed they had done so. When I succeeded in doing it, he became sensible of the difference; and I desired him, on his taking leave, always to use in future a No. 12 catheter, with a very round point, that the passage of urine through it might convince him of the fact." 27.

In the third stage of the disease, when much difficulty of voiding the urine is experienced, many serious symptoms are the consequence. To two of these Mr. Guthrie draws attention.

The first results from the occurrence of pouches in the vesical parietes. Such a pouch having formed, will probably increase, and, having thus increased, may perhaps become the seat of a calculus. Whether the latter be contained in it or not, urine is collected, and in some measure confined. The symptoms of this occurrence, though various and ambiguous, are deserving of attention. In a case that occurred to Mr. Guthrie, after emptying, as he supposed, the bladder by the catheter, he found that he could get a further quantity of urine by passing the instrument in a certain direction; probably it entered a vesical pouch. If the bladder be emptied by the catheter in the erect position, and the patient be made to change it by lying down, retaining the catheter in its place, an additional quantity may run from the instrument, showing that one or other of these pouches has been emptied.

"A gentleman consulted me on account of a difficulty he had in passing his water, for which he used an elastic catheter twice a-day and sometimes thrice, with great relief. But the symptom he complained of most, and for which he applied to me, was, that after emptying his bladder in the erect position on going to bed, he soon after felt as much desire as before to make water, and that on straining forcibly he could pass a little. I desired him to use the catheter a second time when he felt this uneasiness, which he did, and obtained about three ounces more water. This led to the belief that he had pouches in his bladder, which were only emptied by change of position. I wished him to ascertain what position emptied them, but he could not do this in a satisfactory manner, for a reason which appeared after death, namely, that there were several pouches, which could not all be emptied by the same position. He obtained considerable relief by first drawing off his water in the erect position, and then by lying down with the catheter in the bladder, and by changing his position from either side to his face (for these pouches rarely form on the fore part) he removed a further quantity, after which he obtained rest, until the pouches and the bladder were refilled, and the desire to discharge his water again became considerable. If this investigation be made in the morning on rising, it does not give so accurate a degree of information, and must not be mistaken for a similar symptom, about which you may be consulted by persons who have no particular disease. It often happens, that at about fifty years of age the urine passes with less force and more slowly than formerly, although there may not be any perceptible or discoverable disease of the neck of the bladder. It has perhaps only lost a slight portion of its elasticity. If the person be very attentive to himself, he finds that he makes his water very well on rising in the morning; and that during the time of dressing, he makes it once or twice in larger quantity. This may happen to any one at any age, and arises from the circumstance of the urine not being secreted usually to any extent during sound sleep, whilst the kidneys become after such state of rest, much more active on the person's moving about. It is not a sign of disease. The urine is also devoid of any offensive smell, which is rarely the case when it has been retained in pouches. In one gentleman, the existence of one or more pouches of this kind became evident on injecting the bladder; twelve ounces of warm water could be thrown into it before much uneasiness was produced; but on drawing it off, ten ounces only could be obtained, and rarely the whole twelve even by any change of position." 30.

These facts and observations are highly deserving of attention. It is the recollection and the application of many, and sometimes of trivial circumstances, that constitute superiority of judgment and of tact in the diagnosis of disease.

The second symptom to which Mr. Guthrie draws particular attention, is what he denominates the "fluttering blow of the bladder." This blow, inflicted on the catheter or sound, has sometimes been mistaken for the impulse produced by the contact of an instrument with a calculus. Mr. Guthrie appears to have met with four cases in which the circumstance occurred, and from those cases he deduces a description, and attempts an explanation of it.

The first case was that of a soldier in the York Hospital, invalided for some complaint of his urinary organs. He suffered from stricture, but when this had been removed, the symptoms were not materially mitigated. On examination with the catheter, a smart blow was felt on the instrument with the termination of the flow of urine, giving rise to the idea of a stone. This always took place, and sometimes the stroke seemed to be repeated twice, or even three times, although each time fainter than before. The first blow would sometimes force the catheter from between the finger and thumb, when slightly held, and at least two inches out of the urethra. The case was supposed by some to be stone, but the difficulty was never dispelled by an inspection of the parts after death, for the patient was discharged.

In the second and third cases the sensations were the same, save that the little taps on the catheter resembled more the blows given by the wings of a bird in fluttering.

The last case afforded a solution of the symptom. The blow was perfect, but unattended with the grating which a calculus occasions. Mr. Guthrie examined the bladder several times very carefully, and allowed the urine to flow between the blades of the small calculus-forceps, which were kept apart to receive any thing which might fall between them. The silver catheter often received so smart a shock, that it was forced out a couple of inches, and from between the fingers when held loosely, so that the patient himself could not help observing it, and asking the cause. The patient died. There was not a stone of any kind, and nothing peculiar, save five pouches, and "the bar" at the neck of the bladder, formed by its elastic, but now rigid substance, totally unconnected with the third or middle lobe of the prostate. The "fluttering strokes" of the bladder on the catheter were caused, Mr. Guthrie entertains no doubt, by the descent of the pouches containing urine, which, being more or less solid substances, fell, or were forced against the instrument, by the muscular efforts of the bladder in contracting, to effect the expulsion of the last drops of urine.

"When symptoms have given rise to the suspicion of the existence of a stone, and the fluttering strokes or blows on the instrument have been felt, I suspect it may have happened that the operation has been performed and no stone has been found. At all events, a surgeon may be forgiven for the mistake more readily in this case than in most others, and I therefore dwell upon it longer than it perhaps requires, in order to prevent such a misfortune from occuring in future. I cannot help thinking it has been the most common cause of such an accident, and when I hear and have heard, that although no stone was found, the patient, when he survived, was much the better for the operation, I am more satisfied that I am likely to be correct in my supposition. The good done by the operation was caused by the division of the bar at the neck of the bladder, and the consequent removal of the obstruction to the passage of the urine out of it; and I approve of a proper operation being done for this purpose in certain and peculiar cases to be hereafter noticed, but then the patient is not consenting to an operation, supposing it to be for the removal of a stone, and the surgeon is not relieving him by a mistake." 33.

The attention of surgeons has been greatly directed, of late years, to the causes of death after the performance of lithotomy. The old and the prevailing notion, that patients usually die of peritonitis, has been shaken, if not absolutely overthrown, by pathological investigations, more particularly by those of Sir Benjamin Brodie. Those researches have proved, that diffuse inflammation of the cellular tissue of the pelvis and abdomen, is more frequent and more fatal than genuine peritonitis. The former indeed, very seldom attains a considerable height, without giving rise to more or less of the latter; but cellular inflammation is the primary complaint, and prevention of *it* is prevention of all.

The mode, then, of obviating the occurrence of diffuse cellular inflammation has naturally been a subject of anxious consideration. There appear to be two parties in the surgical world, entertaining two different, indeed opposite sentiments. The one maintains that dilatation of the capsule of the prostate gland is preferable to its division. Sir Benjamin Brodie may probably be deemed the most able supporter of this opinion, which ranks among its advocates the illustrious Scarpa. The other party affirms that division of the prostatic capsule, and of a portion of the neck of the bladder, if necessary, is attended with less risk than forcible dilatation. Mr. Fletcher, of Gloucester, may be looked on as the recent Coryphæus of this sect. Mr. Guthrie would appear to agree with those, who consider inflammation of the cellular tissue as the usual source of danger and of death; but he does not agree with those who suppose that division of the prostate occasions the inflammation. In speaking of the veins which bound the prostate, he remarks as follows:—

"My principal observations on these points will be directed to show, that the infiltration of urine, and which I am disposed to say is the principal cause of death, does not prove fatal from its having been caused by a complete division of the prostate, or of a portion of the bladder beyond it, but from its being penned or dammed up, after it has been allowed to escape, by the levator ani muscle, and the deep fasciæ of the perinæum. It is the want of a proper division of these parts that is the real cause of death, and which takes place, therefore, from a defective manner of performing the operation." 35.

Thus we see another theory of the operation proposed, and another suggestion added to the many which now exist, embarrassing, perhaps, rather than aiding the lithotomist.

For our own parts, we cannot avoid the suspicion that, in spite of all precautions and of all improvements, diffuse inflammation of the cellular tissue must continue to be a frequent consequence of lithotomy. That tissue is remarkably prone to inflammation in all parts of the body. Slight injuries occasion it as well as severe ones, and a trivial wound in the arm or in the leg is frequently followed by cellular inflammation, altogether disproportionate to the violence inflicted. This peculiar tendency to inflammatory action is essentially due to the nature of the tissue—to its great diffusion, its various situations, and the free communication of its several parts and of its individual cells. If a wound in an extremity penetrate the fascia, and affect the cellular tissue below it, the latter is particularly apt to inflame. Effusion of serum, of lymph, and of pus, takes place below the fascia and throughout the limb, and sloughing of the subfascial tissue in the first instance, followed by death of the fascia and integuments, is the natural and inevitable consequence, if incisions fail to arrest the mischief.

The anatomical disposition of the cellular texture of the pelvis and the perineum, is remarkably favourable to the development and diffusion of inflammation of its substance. The fascia of the perineum binds it down externally, and confines the effusion of serum and of pus, which it also directs to the scrotum and abdomen. The pelvic fascia might contribute to protect the cellular tissue of the pelvis which it incloses, but unfortunately that fascia is necessarily divided in the operation of lithotomy. The wound of the gorget or the knife, and the bruising occasioned by the forceps and the stone, must, especially when aided by the contact of the urine, give rise to some inflammatory action in the cellular tissue of the pelvis and the perineum. The inflammation thus necessarily excited must, in some instances at all events, diffuse itself throughout the cellular tissue within the pelvis, and through that also which passes upwards to the spine and loins, behind the peritoneum. We are more disposed to wonder at the rarity than the frequency of the occurrence.

We would not be understood to discourage attempts to diminish the fatality of lithotomy, by variations in the nature, the direction, or the extent of the incisions. Yet we cannot avoid expressing our fear that little remains to be accomplished in these matters. We are afraid that the mortality consequent on the removal of stones of any size from the bladder by lithotomy, will never be much less than it is at present, and we ground this belief on our knowledge of the general and marked disposition of the cellular tissue to inflame. We believe that the improvements yet in store, and such, we cannot doubt, there are, must be looked for in the increased facilities of recognizing the existence and effecting the removal of small calculi from the bladder, and, probably, in the better application of lithotrity.

The conclusion of the second lecture in the work before us is devoted to the description of the—female prostate. The vulgar suppose that the fair sex are usually deficient in this gland, but our author assures us that the ladies are belied, and that they really enjoy the organ in question.

"If the word prostate," says the champion of the rights of the fair, "be used with reference to its derivation, as standing before the vesiculæ seminales, certainly a woman has not a prostate, because she has no vesiculæ seminales, but if it be used as a substantive word, to express a particular thing, in the same manner as the words arteria innominata are now used, as a name for a particular artery, which formerly had no name, then a female has a prostate, for there is a substance of the same shape, form, and nearly of a similar structure surrounding the commencement of her urethra. It is the size of the prostate in a boy before the age of puberty, and resembles very nearly in external appearance the same part in the male." 35.

The prostate gland of the female has no ducts. Mr. Guthrie, indeed, is unwilling to honour her with the possession of a gland, but contents himself with simply awarding her a prostate, or rather a "corpus globosum." With becoming diffidence he assigns the property conditionally only, "until further orders from the critics."

If we hesitate to pronounce an opinion upon these anatomical points, it is because we are aware that Mr. Guthrie has conducted his anatomical researches with minuteness and with care. Without equal attention and examination it would be idle in a reviewer to confirm or to condemn.

The third lecture is occupied with the description and the diagrams of muscles which Mr. Guthrie has discovered around the membranous part of the urethra. Without those diagrams a description would be incomplete, and we can only present the following notice of the muscles alluded to, a notice contained in an account which Mr. Guthrie gives of two preparations.

One shews the membranous portion of the urethra surrounded by a well-defined muscle.

"On the upper part there is a central median line of tendon, which runs backwards to be inserted into the fascia covering the upper surface of the prostate, and again forwards on the urethra through the triangular ligament to be inserted in front of it near the union of the corpora cavernosa. On the under part a similar tendinous line is to be observed, which is attached backwards to the fascia underneath the apex of the prostate, and forwards to the central tendinous point in the perinæum. The muscle on its upper surface is covered by fascia descending from the pubes which adheres to it, and this I take to be what Mr. Wilson described as the tendinous origin of his muscle, and from which he supposed the fibres descended to surround the urethra, which they really do not. From the median tendinous line in the upper part of the urethra the fibres pass outwards on each side, converging towards the centre, where they form a leg, as I term it, of muscular fibres. On the under surface the same thing takes place, and a leg on each side being thus formed from the superior and inferior fibres running from each half of the urethra, they pass outwardly, that is transversely across the perinæum, to be inserted into the ascending ramus of the ischium a little below its junction with the descending ramus of the pubis on each side. These attachments are cut off in this prepartion, but when I pull on each of these legs you see that they surround the urethra like a sling." 41.

For a more complete delineation of the muscle we refer the reader to the work itself. We therefore proceed to the fourth lecture, in which the anatomy of the membranous part of the urethra, and the mode of introducing instruments into the bladder are carefully discussed.

The length of the urethra has often been an object of particular, if not always of profitable attention to anatomists and surgeons. Mr. Guthrie ridicules the attempt to attach much importance to such measurement, the urethra of one person differing greatly in point of extent from that of another individual. In proof of this point, Mr. G. displayed on the lecturing table two urethræ, one of which was eleven inches in length, while the other was but eight. The difference depended principally, of course, on the pendulous portion, or that contained within the penis. Yet the other portions exhibit varieties in various individuals.

"I place no reliance whatever on the measurement of the urethra made after death, for, although the urethra may be then eleven inches long, as in the preparation before you, I never met with a case, unless there was a diseased prostate, in which a catheter ten inches long was required to draw water: on the contrary, one eight inches long will generally be found sufficient, and I have known one of seven answer well; the additional inches being usually gained by the elongation or stretching of the parts during life or after death; and if a surgeon calculates inches as his instrument proceeds, instead of considering the points of attachment as so many land-marks to guide his progress, he will be frequently in error, and always liable to do mischief. I believe the mistake of most consequence takes place in regard to those two parts which are called *membranous* and *prostatic*. In the preparation before you, in which the urethra is eleven inches long, the length of these two portions is near three inches. From the orifice of the bladder to the triangular ligament, the usual length of the prostatic portion is estimated at about fifteen lines, the membranous at about twelve, or something more than an inch for the prostatic portion, and perhaps a little less than an inch for the membranous. In this very urethra, eleven inches long, I have no doubt but a catheter nine inches in length would easily have drawn off the urine during life. I am satisfied that one of eight inches would have done it, and the membranous and prostatic portions, which now appear to be near three inches in length, would have been cleared by an instrument very little more than two ; and this would occur from the manner in which these parts are supported and maintained against the pubes by the fasciæ, which attach and connect them and the surrounding parts to each other. When the prostate is diseased, on the contrary, the urethra at this part is made to undergo a considerable degree of elongation, and a catheter under such circumstances, should be longer and larger in the curve." 54.

Perhaps it may be new to many of our readers, that the glands of Cowper are present in the female, and open, of course, into the vagina. The older anatomists were acquainted with the fact, but probably most of the moderns have forgotten it.

Mr. Guthrie proceeds with the anatomy of the urethra. The student may peruse his pages with advantage; we can only cull a passage here and there.

The orifice of the urethra is in most individuals the narrowest part of the canal. Not only is it narrower than the other portion, but a dense and peculiar structure surrounds it, which renders it little capable of dilatation. If that structure be destroyed by ulceration, the aperture displays in general a great tendency to contraction, and a stricture of the very orifice is formed. We have seen, however, several instances of syphilitic sore of the orifice, which have destroyed the ring at the orifice of the urethra, and have yet induced no tendency to contraction during cicatrization. We lately saw a gentleman in whom a circular venereal sore had scooped out about a sixth of an inch of the substance surrounding the orifice. The ulcer had healed, but a patulous aperture of good dimensions remained. Generally, the fact is as Mr. Guthrie has stated it, and the disposition to contraction is productive of annoying or even of distressing consequences. It is not uncommon for patients to apply at the Lock Hospital, with the orifice completely obliterated by cicatrization, and the urine voided by one or by several small tortuous channels opening on the inferior surface of the glans, or even farther back upon the penis.

The orifice of the urethra is sometimes very small as a congenital formation. Sometimes this is unaccompanied with any other apparent aberration from the natural condition; frequently a kind of valve, consisting of membrane, and extended across the lower portion of the aperture, is the cause of its diminished volume. In either case division is often indispensable. This Mr. Guthrie does with a small, sharp, and strong iris knife, carried directly downwards. The wound is apt to heal if care is not taken to keep the edges apart by introducing a bougie from time to time to separate them.

Mr. Guthrie's directions for passing an instrument into the bladder are circumstantial and judicious. But their character is too elementary to render a further notice of them necessary. We may content ourselves with stating that Mr. Guthrie impresses on the mind of the student the rule of keeping the point of the instrument sliding against the upper surface of the urethra ; and, in cases of enlargement of the prostate gland, he insists on the employment of a long large catheter, with a considerable curve. He glances at the occasional perforation of the prostate by the catheter, a perforation generally accidental, seldom injurious, and sometimes serviceable.

It is better that the prostate should be perforated by an instrument than that puncture of the bladder should be had recourse to; but, of course, it is still better that where neither is required neither should be done.

The fifth lecture is exclusively dedicated to observations on catheters and sounds, and to comparisons of their respective merits and value. This lecture, though useful to students, we may disregard, and proceed to the consideration of the sixth, in which a moot and knotty point, the structure of the walls of the urethra is taken up by Mr. Guthrie. The opening of the lecture resembles the apostrophe of Pilate. "What is truth?" said the Roman governor;—"what is the urethra?" says the English doctor. Both questions are more easily asked than answered. The metaphysical analysis of truth might seem more calculated to occasion doubts than the physical examination of a human organ. Yet the Sorbonne might probably fail to produce more eager disputes or more angry disputants in one instance, than the schools of anatomy have done in the other.

Mr. Guthrie endeavours, like some able umpires, to compose the quarrel, by pronouncing all the parties in error. He neither agrees, in the case of the urethra, with those who affect to find it muscular, nor with those who are content with deeming it elastic. But he passes in review its various parts, anatomises their structure, and investigates their functions. He observes that all parties appear to him to consider the wall of the canal to be similarly composed in its whole length: and he apprehends this to be a source of error, there being three principal divisions very differently circumstanced with respect to the surrounding parts. Those divisions are the prostatic, the membranous, and the bulbous and spongy parts of the canal.

Mr. Guthrie goes on to state that it is acknowledged by almost all surgeons of experience that the prostatic and membranous parts are not usually the seat of stricture, which is confined to the bulbous and spongy parts, opposite and anterior to the ligament of Camper. We conceive that Mr. Guthrie labours under a misapprehension on this point. In one of the latest works upon the subject, the lectures of Sir Benjamin Brodie on diseases of the urinary organs, that able surgeon states that—"The ordinary situation of a permanent stricture is at the anterior extremity of the membranous part of the urethra, just behind the bulb of the corpus spongiosum."* Mr. Guthrie, however, is opposed to Sir B. Brodie on this question of fact, and affirms that the bulbous and spongy parts of the urethra are the ordinary seat of permanent stricture. It it necessary to state this circumstance explicitly, because it is the peg on which Mr. Guthrie's argument is hung.

That argument may be reduced to the following positions:—that as the membranous part of the urethra, surrounded and compressed by muscular fibres, is not the ordinary seat of stricture, it is probable that the existence and the action of muscles are not materially operative in its production. It is true that at the bulb the accelerator urinæ is found, but it is true likewise that stricture may exist in the anterior part of the canal, where no satisfactory muscular apparatus can be seen. Mr. Guthrie finds in the corpus spongiosum a more efficient agent both in the production and perpetuation of stricture.

---

* Vide Opus. Cit. p. 6.—*Eds.*

"The whole anterior portion of the urethra, or that part in which stricture is usually situated, is surrounded by the corpus spongiosum; and it is to it that I am disposed to attribute the principal share in the formation of the worst kinds of permanent stricture, and the great difficulty which is experienced in effecting a perfect or radical cure." 76.

It is evident that the truth of this theory of stricture, if theory may be deemed an appropriate term, must stand or fall by the accuracy of the fact on which it is erected. That fact is the greater prevalence of stricture in the spongy than in the membranous part of the canal.

Mr. Guthrie dwells on the elasticity of the corpus spongiosum in its healthy state—on its inelastic hardness where the seat of stricture. When the spongy body is erect, the hardened portion continues undilated, whilst the rest is distended before it and behind it. If the hard part be cut into, the corpus spongiosum seems to have lost its spongy appearance, its erectile texture has become consolidated, and resembles rather a solid gristly substance than an elastic structure. This kind of disease is very apt to form when the urethra is ruptured, during the severity of what is termed a chordee. The preceding account of stricture in the corpus spongiosum is equally consistent with reason and with fact. The statement which succeeds we do not so clearly comprehend.

"When the mucous membrane is inflamed, and has lost part of its elasticity, it does not always yield as readily under distention as some of the interstitial parts of the corpus spongiosum; which when they give way, allow the blood to be effused in the strict sense of the word; and a soft swelling takes place, which is a sufficiently remarkable although not a very common accident. I have just now under my care a young gentleman, who has a soft swelling of this kind about two inches and a half from the orifice of the urethra, and which appeared suddenly. The urethra was inflamed at the time, but was not ruptured; a full-sized bougie could and can be readily passed along it. It gradually altered its appearance, became less, and would I think have gone away altogether, had not another gonorrhœa supervened, which by adding new symptoms, has rather increased than diminished it, and without great care a stricture will possibly be the result. In a case which I treated many years ago in the York Hospital, the swelling situated in the same place was as hard and as circumscribed as if a Barcelona nut had been inserted into the under part of the urethra. It was quite cartilaginous to the touch, and the man made his water almost by drops. I removed this disease by the repeated but careful application of the argentum nitratum, so that no signs of it remained externally, the hardness having gradually diminished until it went entirely away. The man, a soldier, was to have been discharged, but on leaning over his bed to fold up the blankets one morning, he fell forward dead. I opened him next day, and found his heart diseased. The urethra appeared quite sound, and to my great surprise nearly as much so at the part which had been affected as any other. I had a preparation made of it, and it is or ought to be in the museum at Chatham. Lest you should go away with the impression that a stricture of this kind may always be cured by caustic, I must mention to you the case of a gentleman who consulted me a short time afterwards. He had a similar swelling situated at the part where the scrotum joins the penis. I was delighted to have the case, and felt assured of a similar termination, but no such thing took place; the swelling and hardness increased rather than diminished, and at last I was obliged to divide the part from without inwards to save his life, by giving free passage to his water. I have since had many other cases of a like nature, all of which have been treated and relieved with various degrees of success, but none so pre-eminently well as the first.

You will ask me, perhaps, why? I can only say, it is as difficult to answer you on this point, as it is to tell you why in some cases of almost impermeable stricture, a permanent cure is effected by simple dilatation, whilst in others as nearly alike as possible, the relief obtained is only temporary. It depends on the various shades of distinction between diseases, and on the particular extent to which each peculiar structure is affected. Experience, assisted by careful observation, may enable us to select the best and least dangerous mode of treatment, but it has not as yet enabled us to mark all the distinctions and shades of difference between these diseases, which it is necessary we should know to arrive at a more perfect knowledge of their treatment." 81.

It is not evident, at all events to us, what the tumor to which Mr. Guthrie alludes is actually composed of. He gives but one dissection, and in that he found nothing. Does Mr. Guthrie wish us to suppose that blood is effused into the corpus spongiosum, that it constitutes a soft and circumscribed swelling, that this, by some change in its component parts, afterwards grows hard, and becomes a source of stricture? This appears to us to be the gist of our author's observations and description. Yet this is attended with some little difficulty. In the first place, it has not the demonstration of dissection—in the next, it does not seem easy to suppose that effused blood should form a limited swelling, in a cellular part like the corpus spongiosum. But, perhaps, we misconceive Mr. Guthrie's meaning; certainly we must own we never saw a case of the nature he describes. We have witnessed many instances of circumscribed swelling connected with the inferior part of the urethra, from its external orifice to the scrotum. But these swellings appeared to be abscesses, either connected with the lacunæ or dependent upon stricture.

Mr. Guthrie guards, with the utmost caution, against the imputation, that he limits stricture to the spongy part of the urethra. He merely affirms its preponderating frequency of occurrence in that portion. He approaches, and he does no more, a question of some delicacy and considerable difficulty —the exciting cause of stricture.

"The loss of elasticity of these parts is to be accounted for, and my belief is, that it is caused by inflammation in all its shades and stages. In this I support the opinions of Sir C. Bell, Mr. Shaw, and others, against those of Sir E. Home, Mr. Wilson, and those authors who have directly or indirectly taken a different view of the question; and attributed the contraction to a wrong action of muscular fibres which have not been satisfactorily shewn or proved to exist. The most remarkable fact on this point I have stated, *viz.* that in the membranous part of the urethra, which is known to be surrounded by a very powerful compressor muscle, the advocates for muscular contraction admit that permanent contraction does not usually take place; and if it does not take place there, it is and will be difficult to prove why it should occur from that cause anywhere else." 83.

In the next lecture, this question is considered more at length.

It is considered more at length, yet we do not suppose that Mr. Guthrie will feel mortified when we own that he does not satisfactorily decide it. After briefly exposing the opinions of Mr. Hunter, Sir E. Home, and Sir C. Bell, Mr. Guthrie observes that he has met with one case, and one only, of spasmodic stricture.

"The only case of pure spasmodic action, which has come under my observation, occurred in a gentleman, who has come to my house twice, in the course of several years, declaring he could not make his water, and desiring to have the catheter passed, which was each time done without the least difficulty. The first time he came he was quite aware of his situation ; said it arose from anxiety of mind relating to family affairs, and that the passage of the instrument would immediately and effectually relieve him. If there was any obstacle, and I was by no means certain of there being any beyond a hesitation, it was at the commencement of the membranous part of the urethra, and arising, I suppose, from a spasmodic contraction of the compressor urethræ of Mr. Wilson, of which I have given a detailed description. As this gentleman suffered no kind of inconvenience at any other time, I am induced to believe, that there was no particular irritation in the urethra, and that it was, as the cause is unknown, what may be called accidentally spasmodic. I have read of a lawyer, a gentleman, but I do not recollect where I saw it, who often suffered in this way when engaged long in court in a difficult case, and who was always relieved in a similar manner ; but here I should say it is more than probable the individual was labouring under some slight permanent irritation in the urethra, or that it was at least in an excitable state at some one part near the bulb." 88.

Mr. Guthrie remarks, that the more common cases of what is considered spasmodic stricture are those of young men, who, when suffering from gleet or gonorrhœa, commit an excess in some stimulating liquid. Retention of urine, with violent straining and excessive agony ensues.

"These (says Mr. G.) are called cases of spasm, I call them cases of inflammation, and which induces a want of consent, as Sir Charles Bell expresses it, between the muscles of the parts, so that when the bladder acts, the muscles surrounding the urethra act by yielding and dilating as they ought to do, but remain, or become more permanently contracted; the urine is forced against the inflamed and contracted part of the urethra, and by its irritation increases the mischief. When the water is drawn off, the desire to pass it is removed, and the greatest irritation on the inflamed or irritable part of the urethra is thus taken away. Experience has also long pointed out to us, that when the patient can pass his water, the complaint is yielding, and the object is to get it to flow, and mechanical means in these cases will always do more than general ones, although I by no means deny their use as auxiliaries. If the case be more advanced, and the catheter will not pass, or you are at a distance from home and have not one small enough at hand, take a common bougie, and soften the point by dipping it into warm water, but which is not warm enough to melt the material of which it is composed, pass it down to the obstruction, and press it steadily, but not painfully so, against it, and let it remain for several minutes. It will often be found to pass on, or the patient will find on withdrawing it that he can pass his water in small quantities. The mischief here is a slight degree of inflammation, aggravated by cold or intemperance, but without any permanent alteration in the structure of the urethra, yet I do not believe it is caused by spasm." 91.

The experienced critic will too readily perceive that Mr. Guthrie asserts what he does not prove, and denies when he does not explain. We cordially agree with Mr. G. in believing, that what are considered cases of spasmodic stricture are really instances of inflammatory action, invading the urethra. But how does inflammation immediately give rise to retention of urine? Surely it must be by exciting spasmodic contraction of the muscles. No other physical alteration in the canal could appear so suddenly and act so perfectly.

Mr. Guthrie rejects the agency of spasm, but invokes the same spirit in another shape. The muscles are not spasmodically contracted, but "a want of consent exists between them." The term is elegant; but how does Mr.

Guthrie know that such discord distracts the muscular system? Besides, it implies no positive and obstinate contraction of one muscle, or one set of muscles; and yet such a positive contraction there must be, to render the whole power of the bladder nugatory in propelling the urine through the urethra. If, however, a want of consent means this, we submit that it is only another designation for the self-same thing. For our own parts, we entertain little doubt that the muscles surrounding the bulb, or the membranous part of the urethra, are in some cases spasmodically affected. The history and all the circumstances go to prove it; and we have seen the bougie, introduced into the strictured part, convulsively grasped, and even jerked, by the violent action of the muscles. At the same time we repeat our belief, that inflammation is a much more frequent cause of the spasm than is usually supposed, and we feel convinced that permanent stricture frequently results from the inattention of the surgeon to its inflammatory origin.

It is difficult to collect the opinions of Mr. Guthrie on the real nature of that temporary obstruction, which he feels disinclined to attribute, in general, to spasm. But the following passage is, perhaps, the most apposite we can select.

"I am led to infer, from a due consideration of these and many other similar cases, that the canal of the urethra may be perfectly closed for a considerable length of time by a spasmodic contraction of its muscular coat of a transitory kind, provided such muscular coat were believed to exist; but as such belief is not commonly entertained, I prefer supposing that the obstruction takes place from inflammation of the internal mucous membrane, which alters its attractions and properties, assisted by an undue contraction of the elastic and outer wall of the canal, dependent on its vital elasticity or contractibility. I believe this to be the case in all instances except those alluded to in the commencement of these observations, in which an undue action of the compressor urethræ muscle alone may have produced the effect." 100.

We are not much inclined to argue questions mainly hypothetical. But a more pugnacious reviewer might urge that, as Mr. Guthrie is willing to believe in the existence of spasm, provided a muscular apparatus be admitted; and, as spasmodic stricture is seated in a portion of the urethral canal, where muscles are actually demonstrable, the affirmative is virtually allowed by Mr. Guthrie. We will pass to the inspection of a case.

"That a muscular coat or wall of a canal can exert an especial and long-continued influence upon it, I have had opportunities of seeing; and in one case in the œsophagus in a very remarkable manner. The patient, a young lady, had suffered for years from repeated difficulties in swallowing, which at last became positive obstructions, preventing the passage of either solids or fluids for days. The obstruction usually yielded almost suddenly, and the lady could then swallow liquids and small masses of food with tolerable ease. A good-sized œsophagus bougie could then be passed with little sense of opposition, although the stricture was distinct, when swallowing was impossible. It was situated about an inch below the situation of the cricoid cartilage, and no bougie could then be forced through it, although frequently attempted by several very able men. As the complaint continued, the impossibility of passing a particle of food became more frequent, and lasted for eighteen, twenty, and six and twenty days together, so that at last the lady became quite exhausted, and died from inanition, in the full possession of her senses, *and with a Christian resignation of so perfect and admirable a nature, that it was impossible to look upon it but with the strongest feelings of gratitude to God for his goodness.* On examination I found the œsophagus externally of its natural appearance, without the slightest sign of constriction. When slit open, it appeared of its usual thickness, and without any deviation from its ordinary state with respect to the appearance of the muscular layers; but on the inside, and adhering firmly to the mucous coat, there was a false membrane, the upper edge of which appeared to have been separated, in consequence of the repeated application of the bougie, and a little turned inwards, so as to fill up, in part, the canal, through which, however, any common-sized bougie could after death be passed. The mucous membrane from this part onward to the stomach, which was not allowed to be examined, seemed to have lost its normal character, and to have taken on that of a serous one on which a false membrane readily forms, but which rarely occurs on a mucous one, unless some great alteration has previously taken place in it. The difficulty which existed at all times, for several months, arose from this false membrane, which could be peeled off, and resembled chamois leather; whilst the permanent and insurmountable obstacle, which often occurred for three weeks at a time, must have arisen in part, I conceive, from muscular contraction, although no trace of permanent stricture was observable after death." 99.

The preceding case is highly interesting, and rather curious. It appears difficult to suppose that an obstruction could depend, for such lengthened periods as three weeks, upon muscular contraction. There is another supposition, uncharitable we admit, but not utterly devoid of probability. It may be guessed from the mention of a case.

A female was attended by one of the most dexterous surgeons in London. She was said by him to labour under stricture of the œsophagus, through which no bougie, large or small, could be passed. After an attempt to introduce one, the patient was attacked with symptoms of inflammation of the lungs, and died in the course of a few days. On examination of the body, it was found that no stricture of the œsophagus existed. The extremity of the bougie had lacerated the mucous and cellular tissues of the œsophagus, inflammation of the external cellular tissue had resulted, and extending along the posterior mediastinum it had reached and affected the lung of the left side. We have heard of other not very dissimilar cases, and we entertain no doubt that stricture of the œsophagus is frequently thought by able surgeons to be present, when the instrument is impeded by other and more natural obstructions.

We have taken the liberty of placing in Italics a particular passage in Mr. Guthrie's narration of the case. It is that which refers to the edifying resignation of the patient. Such passages are strongly indicative of the piety, but not so conclusive of the judgment of the author. For the sceptic may perversely and ingeniously hint, that the goodness of God would have been carnally more manifest, in withholding the *cause* of misery and suffering, than in granting to the sufferer firmness to endure it. Yet the influence on Mr. Guthrie and on others was so salutary, as probably to compensate for the apparent evil.

We must pass over Mr. Guthrie's notice of permanent stricture of the urethra. We have hitherto considered little more than a third of the interesting volume before us. Our glance at the remainder must, we fear, be more hurried than its merits might demand.

The symptoms of stricture are well described. But they are familiar, and we need not reiterate them here. We shall merely draw attention to one circumstance.

"The testes at an early period of prostate irritation often partake of disease. This has often been supposed to depend on sympathy, but I apprehend, and am inclined to believe with Mr. Abernethy, that it occurs from the continuous propagation of irritation from the opening of the ejaculatory ducts into this part of the urethra. The testes become uneasy, then painful, and a little swelled. In warm climates the irritation usually terminates in hydrocele, with a softened and enlarged, or sometimes a hardened and enlarged testis. In more northern climates, abscess occasionally takes place, or chronic induration is sometimes mistaken for scirrhus." 109.

The progress of pathology has done much towards routing the locust host of maladies depending upon "sympathy," and "irritation," and abstractions of a similar description. We would not be understood as denying that such things as sympathy and irritation exist. But there cannot be a doubt that the terms were much abused, and that actual and tangible organic alterations have been frequently neglected, in the loose and inexact philosophy that delighted in their liberal admission. Inflammation of the testis, in gonorrhœa, has often been described, and is constantly believed, to result from sympathetic irritation. Yet surely, when we see that a continuous tube connects the testis and the seat of gonorrhœa, it is much more natural and much more easy to believe that inflammatory action extends along it, than that some obscure and hocus-pocus process sets up disease at two distant points.

It is proper to advert to Mr. Guthrie's mode of examining the urethra, and the plan which he adopts for the cure of stricture.

In a young man, who makes his water in a tolerable stream, Mr. G. employs for the former purpose a solid silver sound, not more than two-thirds of the size of the canal. When a moderate-sized instrument cannot be passed, Mr. G. has recourse to a small bougie; and, if this cannot be introduced, he adopts another process.

"In these cases, it will be necessary to take an impression of the face of the stricture, in order to discover where the opening is situated, by which means the point of the instrument may be more certainly directed to it. This is accomplished by means of a middle-sized bougie, the point of which is first softened by dipping it into hot water, or by a model bougie, the point of which is made of softer materials than the shaft. This should then be well oiled, and passed down to the stricture, against the face of which it is to be gently but steadily pressed. If the stricture, although narrow, is not very tough or permanent, it will sometimes yield, and the softer bougie goes through, when the point of the harder one only bends, twists, or turns back, the bougie having doubled on itself in the passage. If the stricture does not yield, the soft bougie does, and is gradually pressed into the sinuosities or openings on its surface; so that after remaining some minutes it is withdrawn with one or more processes projecting from the end of it, either acute or obtuse as the case may be, and indicating the commencement of the true, and sometimes also that of one or more false passages. The experience of the surgeon will now be his best guide; he will remember that this obstacle is much more often below than above, and that a channel in that direction is much more often a false than a true one." 119.

The seat and the size of the opening being obtained, a duly adapted instrument must be selected, and dexterity or good fortune must conduct it to the opening. If a bougie, it should be allowed to remain in for an hour; and if a catheter, it should be kept in altogether, unless the bladder is peculiarly irritable; and then it need not be carried into it, but be firmly fixed an inch beyond the stricture. This method is not however, necessary, and is often so inconvenient to the patient's pursuits that it cannot be adopted. Such is a brief outline of our author's method of examining a stricture.

The treatment, he observes, may consist of dilatation, the application of caustic, division, or the combination of all these measures. The following is his procedure by means of dilatation.

"As an impassable stricture cannot be cured by dilatation, until such time as a passage is obtained through it sufficient to admit a small bougie, I shall for the present suppose that passage to exist. A bougie, of a size that will pass without inconvenience, is to be introduced and allowed to remain for an hour, and if the bougie is a little conical, the stricture may not only be completely filled by it but moderately dilated. If the stricture be very irritable, the soft bougie may be grasped and marked by it, and the same thing will occur if the bougie be too large and too strongly forced into it. If the bougie be rather too large at the point it will not proceed on meeting with the stricture, although sometimes by a gentle pressure for two or three minutes, the stricture will gradually yield and allow it to go through; but there is a probability that more irritation will follow this mode of proceeding than if a small one is first introduced for a quarter of an hour, and a larger one then made to take its place, which it will almost always readily do. This is in fact the principle on which dilatation should be conducted, and it will always be accomplished more safely and easily for the patient if done in that manner; for a larger bougie can always be introduced if it follows a small one, than can be got into the bladder without such a precursor. This is a fact that does not admit of a dispute. I never use the bougie as a mere dilating instrument oftener than every two days, and when the urethra is irritable only every three, and sometimes four days. Proceeding in this manner the stricture gradually yields, and a bougie, whether made of plaster or of silver, and as large as the orifice will permit to enter, will at last proceed through the whole passage without meeting with any obstacle; and it ought to be repeated at longer intervals until this disposition for contraction seems to be removed. When a stricture has been a permanent one, the patient should be taught the manner of doing it, so that he may use it once a week, then once a fortnight, and at last once a month or quarter." 123.

If the orifice of the urethra is too small to admit of an instrument of requisite dimensions, its division downwards must be practised, a full-sized bougie being subsequently used every morning to prevent contraction during cicatrization. When the orifice has been enlarged, it is occasionally found that an instrument of the necessary size is not well borne by the portion of the urethra not actually strictured. Under these circumstances, surgeons have attempted to invent a sort of bulbous sound or bougie, which should operate exclusively upon the stricture. As a little consideration will shew the inevitable futility of such a scheme, we need not do more than remark, that Mr. Guthrie expended much time and trouble unsuccessfully in endeavouring to carry it into execution.

The destruction of stricture by the agency of caustic occupies the most considerable portion of the next (the ninth) lecture. Yet Mr. Guthrie owns that it is difficult, if not impossible, to apply with benefit the caustic, to such strictures as will not admit of the passage of a bougie; and doubts or denies the greater permanency of cure, when effected by caustic, than when owing to simple dilatation. Although it is true that the whole of the argument is not comprehended in the points affected by these admissions, the pith of it is certainly comprised in them. In short, if Mr. Guthrie be correct, the advantages of caustic must be very slight; for dilatation is necessary at first, when the stricture is most narrow, and necessary at last to obviate its recurrence. There may be, and probably there are, *some* cases in which the application of caustic may be serviceable. But balance the capricious and dubious advantages against the certain and general risk, and the practical scale goes heavily down on the side of danger and disgrace. To drop metaphor; caustic is deservedly rejected in the ordinary treatment of strictures of the urethra. We turn to another subject—the accession of paroxysms of fever subsequent to the introduction of a bougie.

Surgeons are familiar with this occurrence, and the means of preventing it have exercised their ingenuity, though probably without commensurate success. Sir Benjamin Brodie has imagined that the contact of urine with the urethra, after its irritability was excited by the bougie, might occasion the febrile accession. He recommended that the catheter should be retained in the urethra and bladder, in order to allow the urine to escape, without coming into contact with the sides of the urethra. We are bound to confess that we have seen rigors and fever supervene, when no urine was voided in the interval between the introduction of the instrument and occurrence of the rigor; and, in other instances, we have witnessed the latter when a catheter was kept in the urethra. On these facts we can speak with decision. Perhaps in this, as in many other cases, the simplest and most obvious explanation is the true one. Probably the mere introduction of the instrument occasions, by the impression on the nerves of the urethra, the rigor and concomitant phenomena. Mr. Guthrie offers the following suggestions.

"When a paroxysm of fever is about to be produced in consequence of the irritation caused by the introduction of too large a bougie, the desire to pass water is urgent, but the patient is incapable of doing it, and the accession of the cold fit shews the course of the affection. This will be best alleviated by a grain of opium and ten grains of camphor, but the most efficient remedy is to pass a small elastic gum bougie through the stricture and fix it in the urethra for a few hours: it takes off the irritation, and often seems almost to arrest the paroxysm of fever. It is a practice which should never be neglected in serious cases, although it is rather in opposition to prevailing theories." 135.

We must own that we have found the introduction of an instrument more frequently aggravate than prove advantageous in cases of this nature. We lately had a patient, in whom the attempt to retain a gum catheter in the urethra, almost invariably gave rise to distinct intermittent fever. Undoubtedly a full dose of opium will prevent, at least in many cases, the accession of the paroxysm, but sometimes it will fail; and the treatment which usually answers best, is to keep the patient quiet and in bed, and not to persist in the introduction of instruments until the irritability of the urethra is diminished. Patience, and abstinence from violence, are highly requisite on the part of the surgeon. When the fever assumes, as it sometimes does, a distinctly intermitting type, quinine or arsenic becomes as useful as in ordinary intermittents.

The next point to which we would direct attention is hæmorrhage from the urethra. On this head, Mr. Guthrie relates some interesting facts. The most alarming hæmorrhages which he has seen have been, he informs us, "from common causes." He presents two specimens of this description.

*Case.* A gentleman had had a catheter passed by a surgeon of great reputation and ability in the morning, without inconvenience or pain. On his return home he found there was a considerable oozing of blood, which continued during the day, and induced him to send in the evening for his surgeon, who was unluckily out of town; the bleeding increased in the night, and in the morning early Mr. Guthrie saw him. There were several tubs of ice and water in the room, all apparently containing a considerable quantity of blood; his face was deadly pale, the pulse scarcely perceptible. The bleeding was arrested in a few minutes by pressure on the perineum, and did not re-appear.

*Case.* A tradesman passed for himself a common soft bougie, the point of which caught in some part of the urethra, and apparently penetrated into it. Bleeding supervened, and continued two days and two nights, when Mr. Guthrie was requested to visit him. Mr. G. found him kneeling in bed, and straining violently to pass his water, but which came with great difficulty, as the bladder contained a good deal of coagulated blood, which had passed backwards into it. He was as white as a sheet, and fell back in his bed, nearly insensible, almost as soon as Mr. Guthrie entered the room, having, as he said afterwards, passed several quarts of what (as it all coagulated) he considered to be pure blood. The bleeding was permanently stopped by pressure.

"For the purpose of knowing where to make the pressure, any light, flat, and narrow, but firm substance should be prepared, such as a piece of cork, which can always be procured. The patient should then force all the coagulated blood out of the urethra; and as the bleeding usually takes place in these cases from that part which is anterior to the triangular ligament, pressure can readily be made upon it externally; but as it may be made a little before or behind the exact spot, in either of which cases it would be useless, the selection of that spot must be well made. This is done by beginning as far back as possible, and gradually bringing forward the finger by which the pressure is made. At a certain point the flow or dripping of blood will be arrested, and the precise spot from which it comes will be in all probability a little behind where the finger rests, a fact which can also be easily ascertained by carrying the finger a little backwards, when the blood will again flow. The bit of cork or pad can now be duly placed, and the patient should be desired to make pressure on it himself, and which he can often more readily do than an assistant." 138.

When the hæmorrhage proceeds from the neck of the bladder, or prostatic part of the urethra, cold water, rest, and an opiate will arrest it, provided it is not caused by malignant disease. A case in point is given by our author.

*Case.* A gentleman passed a bougie for himself. It was larger than usual; and it caught on some fold at the entrance of the bladder, which it passed with a jerk. A continued bleeding was the consequence, accompanied by an urgent desire to make water, but which appeared to be blood, or nearly so. The desire soon became more urgent, and the difficulty of passing any thing greater, until at last complete retention ensued. In order to remedy the retention of urine, Mr. Guthrie passed a small gum-elastic catheter, which drew off a quantity of bloody urine, and relieved the irritation and desire existing at the neck of the bladder, which soon afterwards subsided. He then directed an opiate to be given, and sent the patient to his bed. Some blood oozed from the urethra, and the patient passed dark-coloured bloody urine for twenty-four hours afterwards, but the bleeding never returned.

Mr. Guthrie alludes to the reflux of blood from the urethra into the bladder, and the consequent plugging of the latter with coagulum. He observes that the proper practice to be pursued in such cases is to inject the bladder with warm water through a catheter with a single large eye on the side and a hole at the end, or by a double catheter, by the motions of which, in the first instance, the large coagulum may be in some degree broken up, when it is more readily dissolved by the water, so as to leave the urine quite clear in a few days, provided no more blood is poured into it.

Mr. Guthrie mentions the particulars of a case in which the hæmorrhage depended on malignant disease of the bladder. The source of the hæmorrhage was evident during life, from the circumstance of its being accompanied with shreds of what resembled medullary matter. He mentions also another case, more interesting on account of the obscurity surrounding the source of hæmorrhage. The patient was an elderly medical man, who occasionally voids dark-coloured bloody urine. The blood was supposed to proceed from the kidney, and various medicines were taken in consequence.

"As some little difficulty seemed to exist on the first attempt to evacuate the bladder, and as the bleeding might arise from the irritation caused by a small stone, it became necessary to examine the urethra and bladder. Nothing could be made out, save a slight difficulty on entering the neck of the bladder with the sound No. 12, and which part offered a positive obstruction to No. 14, surmountable only by a little management of the point of the instrument. He has in fact the bar, or dam, I have pointed out as occasionally forming at this part, independently of any disease of the prostate; and as he now finds that his urine is clear for many days together, and that he can always cause it to be a little bloody, either by passing a large bougie or by a little more than his ordinary exercise, he has acceded to my original opinion, that the blood comes from some enlarged veins at the neck of the bladder. He can now pass No. 14 through it with ease, and is much more free from the passage of blood than formerly. He therefore uses the solid silver sound once a fortnight, and has nearly abandoned that of internal medicine. I was led to believe that the veins of the neck of the bladder were enlarged, first from there being obviously some derangement of structure as well as of function at the part; and from perceiving that the veins of the nose, as well as those of the glans and prepuce were very blue and tumid, appearing as if they did not truly transmit their blood through them: and it struck me that those of the neck of the bladder might be in the same state. The sound only did good, or does good, by preventing the increase of the bar, and thereby rendering an undue action of the bladder unnecessary; the part is in fact at rest." 141.

The tenth lecture is devoted to the consideration of suppression and retention of urine. Of the first we need say nothing; of the second, not much; for the subject has been lately worn till it is thread-bare. We may observe that Mr. Guthrie agrees with those, the best practical surgeons we are sure, who in cases of retention resort at once to the employment of the catheter or the bougie. If these fail, and in dexterous hands they seldom do so, it is then sufficient time to resort to the other and familiar train of remedies of a general description.

The operation of puncturing the bladder or urethra is necessary in the ratio of the surgeon's want of knowledge and adroitness. Yet sometimes it may become inevitable, and the choice of one of the many methods recommended, must present itself to our consideration. Mr. Guthrie prefers the puncture of the bladder through the rectum to that above the pubes. But he seems to prefer the opening into the urethra behind the stricture to either of these plans. We must take the liberty of quoting a passage of considerable length. It is Mr. Guthrie's description of his operation, a description which it would not be well to abridge.

" The patient being placed and secured, as in the operation for the stone, a catheter or sound is to be passed down to the stricture, and held steadily against it, the concavity being as usual upwards, the point directly applied to it. The rectum having been previously cleared by an enema, the fore-finger of the left hand being duly oiled, is to be introduced into it, and the membranous part of the urethra and the prostate are to be examined as well as the bladder, the state of which will in all probability have been previously investigated. If the membranous portion of the urethra is dilated by the urine, so much the better; but the object of introducing the fore-finger is to ascertain the relative situation of the upper part of the rectum and the urethra, which latter part only touches, or is nearly in direct application to the rectum, at the termination of its membranous part and the commencement of its prostatic portion. There is a certain distance, which is greater or less in different individuals, between the last inch of the rectum and the urethra placed above it. The two parts form two sides of a triangle, the apex of which is the prostate, the base the external skin. It is within the two lines of the triangle that the operation is to be done. The surgeon, taking the catheter in his right hand, whilst the fore-finger of the left is applied to the upper surface of the rectum, moves the point upwards and downwards, so as to communicate with the fore-finger of the left hand, and to convey to it a knowledge of the situation of the extremity of the instrument; and particularly of the distance between them, which the motions given to the catheter by the right hand will clearly indicate. The thickness of the parts between the obstruction and the rectum can be estimated with sufficient accuracy, both at the point where the left fore-finger is applied, and at the surface of the skin; for, although the membranous part of the urethra cannot be easily felt from an incision made on the left side of the perineum, it can always be distinguished from the rectum. The next step of the operation is to divide the skin, cellular membrane, fascia, muscular and tendinous fibres, which may intervene between the upper surface of the rectum and the under surface of the anterior and middle portions of the membranous part of the urethra. This is to be done by a straight, blunt-backed, narrow, sharp-pointed bistoury, fixed in its handle, and there are two ways of commencing the operation: the first, when the obstacle is behind the bulb and the external parts are not diseased, may be done by a straight incision, in a perpendicular direction; in which manner the operation may always be done if the surgeon is well acquainted with the anatomy of the parts; but if he is not, or they are very much hardened, and consequently unyielding, a transverse, curved, or crescentic incision should be made across the perineum, the centre of which corresponds with the raphe, and is one quarter of an inch above the verge of the anus, or as near that distance as may be, with due respect to the rectum. This gives room, and allows the parts to be separated as much as they will admit. If the transverse incision is not adopted, the point of the straight bistoury is to be placed on the skin a little above the verge of the anus, the cutting edge being above, the blunt back towards the rectum, the handle being a little depressed, the point a little inclined upwards. The degree of inclination necessary to carry the knife inwards for the distance of an inch, and clear of the rectum, will be indicated by the finger in that part, and the eye of the operator will correspond with the point of the fore-finger, so that the bistoury may be steadily pressed in to that extent, and then carried upwards, and brought out in the exact median line, making an external incision of at least an inch and a quarter to an inch and a half, as regards the external parts; and which may be then extended as space is wanted for the prosecution of the operation. The part being sponged, the surgeon again introduces the bistoury in the median line, the point being directed upwards and backwards towards the urethra, and he may then deepen the cut. The fore-finger in the rectum will always tell him where the back, and consequently where the point of the bistoury is. The opening will now be sufficiently large to allow the operator to lay aside the knife, and to feel for the urethra with the point of the fore-finger of the right hand, an assistant keeping the catheter steady against the stricture, the end of which will now be readily felt. If the point of the fore-finger of the right hand does not go beyond it and touch the sound part of the urethra, which is dilated by the urine in the generality of cases, the knife is to be resumed, and the fore-finger being withdrawn from the inside of the rectum, is to be placed in the wound, on the outside of the rectum, which is to be depressed as much as possible; the back of the knife is then to be turned to it, whilst the point exposes and opens the urethra, and which it can do very easily near the apex or transverse portion of the prostate, or at the termination of the membranous part of the urethra; but it is not necessary to go so far back, and the membranous portion may be opened at its middle with every advantage, and with perfect safety to the gut. A good anatomist and surgeon will open the urethra in this way sooner than the mode of doing it can be described, the urine will make its escape and the patient will be at once relieved. Whether the stricture shall be now divided or not, is a question presently to be considered: but the cure can be completed either with or without it." 170.

We present this proposal without further comment than recommending it to the notice of surgeons. Mr. Guthrie relates no case in which the operation was performed, and we suppose there can be little doubt of the execution being frequently attended with some difficulties.

In the following lecture, the eleventh, Mr. Guthrie considers seriatim the division or non-division of the stricture after the operation of opening the urethra behind it—the causes and the treatment of effusion of urine—and urinary fistula in the perineum. A few words on each of these points may be permitted.

1. Mr. Guthrie is eclectic in his notions and his practice with respect to the division of the stricture. He thinks that division is unnecessary in cases where the stricture is not of any great extent; cases in which the operation of opening the urethra behind it, 'removes the pressure of the urine upon it, and allows it to assume a quiescent and dilatable condition. On the other hand, he is of opinion that the stricture should be divided in completing the operation in all cases in which it is of a thickness, hardness, or extent, leading to the expectation of the cure being difficult or prolonged: for it must be borne in mind, that the incision in the perineum soon closes up, so as to become a very small opening, no better indeed than a fistulous one if the passage is not cleared; and that any particular delay in effecting this object, will bring the parts into the state they are in when a fistula in perineo has taken place from other causes, and which alone often requires another operation of nearly a similar nature for its cure.

The introduction of a catheter into the bladder after the division of the stricture appears to our author to be proper or improper, according to the condition of the bladder. If this organ is irritable or inflamed, the retention of an instrument is dangerous and mischievous. He rather recommends that an elastic gum catheter or a bougie may be passed through the divided part of the urethra into the posterior portion, and retained by tapes in this situation. This effects all the requisite good to the urethra, without the disadvantage of irritating the bladder.

Mr. Guthrie mentions some facts to prove that after the division of stricture from without the patient is exposed to a recurrence of the malady, unless he maintains the calibre of the passage by the frequent or occasional use of a bougie. All practical surgeons are well aware that stricture is seldom permanently cured, whatever the treatment may have been. The advocates, indeed, of particular methods have generally vaunted their power of preventing a relapse. But those acquainted with the subject have listened with a smile to the idle boast.

2. On the symptoms and the treatment of effusion of urine little at this time of day need be said. The employment of scarifications is now universally resorted to. But Mr. Guthrie offers one remark, which, in practice, is perhaps insufficiently remembered. He observes that scarifications are not effectual unless a free incision is made in the perineum, in any way the surgeon may choose according to the nature of the case, until the superficial fascia is fully divided, and a free and direct passage for the urine is obtained; so that there may be no possibility of its escaping into the surrounding cellular structure on account of the dependent outlet which is made for it. He proceeds to recommend that a solid sound or a catheter should be passed down the urethra to the stricture as a guide; and if it be as far down as six inches, an incision is to be made in the perineum, on either side, as may seem preferable, and nearly as in the operation for the stone; this is to be continued inwards, until the finger can distinguish the point of the instrument in the urethra, in front of the stricture behind which is the rupture or hole in the urethra; and the parts must be so divided that the urine will run directly out without further infiltration. He believes that it is always better to divide the stricture if possible, and to carry the catheter into the bladder, from whence the urine may occasionally be drawn off, provided the presence of the catheter is not productive of too much irritation. The last is an important salvo.

3. The profession are familiar with the fact that effusion of urine into the cellular tissue will occasion destruction of the scrotum. But they are not equally aware of the circumstance, that diffuse inflammation not unfrequently invades the cellular tissue of that part, independent of stricture, of urinous extravasation, or of any affection of the urinary organs. The patient is usually debilitated or broken down in health—the disease is commonly preceded by a rigor, or that general disturbance which is generally the precursor of cellular inflammation—it is sudden in its attack, rapid in its progress, and destructive in its consequences. The scrotum swells, and its skin is of a pale or a dusky red—the swelling is of a boggy character, the pressure of the finger occasioning an indentation—the margin of the redness is not so distinct as in the erysipelas, nor has it the same disposition to extend to other portions of the body—after a short period the skin becomes darker and it sloughs—the cellular tissue is then displayed, loaded with lymph, and itself sloughing—and the tunica vaginalis of the testes is exposed. The only chance of arresting the mischief consists in the early employment of incisions. Yet such is, in general, the condition of the patient, that these, however early and boldly resorted to, are incapable of effecting the preservation of the scrotum. The following is the only notice of the malady taken by our author.

"Erysipelas will sometimes attack the scrotum, and simulate the appearance derived from extravasation of urine. I was once called to a case of this kind, in which the scrotum was greatly distended, and I was at first sight disposed to attribute it to that cause, but the patient declared he not only could make his water well, but had never had any difficulty in doing it. I was not, however, satisfied until I had passed a catheter, and assured myself that there was no disease in the urethra, nor in the perineum. The patient recovered, but lost a good deal of the skin of the scrotum by sloughing, in spite of several incisions into it, which saved the remaining portion." 185.

We have mentioned the chief circumstances which disprove the erysipelatous character of the affection. We have had the opportunity of witnessing four cases, and are amply satisfied of its really being inflammation of the cellular membrane of the scrotum.

The twelfth lecture exhausts the treatment of stricture. It is chiefly, we might almost say, totally devoted to the exposition and the advocacy of the method of introducing a bougie or catheter as far as the contraction, and retaining it in the urethra, so that its point may press upon the stricture. The majority of our readers are probably aware that this plan has been strenuously lauded by Dupuytren, and we need not do more than sketch with a light and a rapid pencil the steps of Mr. Guthrie.

It is when the stricture is permeable by the urine, but impassable by the bougie that the latter is employed in this especial manner. Mr. Guthrie prefers a hollow gum elastic bougie, of a medium size, perfectly smooth, and tolerably round at the point, so that it may give as little uneasiness as possible. A very small bougie gives more annoyance than a larger one, is retained with more difficulty, and is more likely to give rise to irritation, in which case it should be removed, and after a little delay, replaced by a larger one. The bougie is to be fixed in the urethra by tapes, and its free end should project rather less than an inch beyond the orifice of the urethra. The point should press against, or rest upon the stricture with the greatest possible gentleness, so that it may not give rise to inflammation or to ulceration, and yet should press just so much as to cause absorption. The nice regulation of the pressure is pleasing to the fancy and apparently consonant with the practice of our author. Pain and irritation must not be excited; occasionally, if necessary, removals of the instrument may be allowed; but the period required for the instrument to penetrate the stricture, varies from three days to six weeks, or perhaps more. Mr. Guthrie has never known the practice unsuccessful. The remainder of the cure, when the instrument has once been passed through the stricture consists in slow and in cautious dilatation. Three cases are detailed; they are interesting and long. Perhaps the short passage which concludes the chapter expresses as much as any other portion of it. It is occupied with the remark that no one plan will succeed in all cases, and that he is the best and most practical surgeon who knows what method to apply to each. With this observation we dismiss the treatment of stricture.

Inflammation, and acute and chronic abscess of the prostate, with low inflammation or irritation of the prostatic portion of the urethra are the subject matters of the thirteenth lecture. Passing over the description of the symptoms and the treatment of acute inflammation of the prostate, we may pause to insert the useful advice of Mr. Guthrie with reference to the opening of prostatic abscess.

"When," says he, "from the continuance of the disease, the occurrence of rigors, and the increase of the febrile symptoms, the augmented sense of fulness and tension in the perineum, and the greater difficulty of making water, the formation of matter may be presumed; an examination per rectum will often give considerable information, in addition to the swelling which may be perceived externally. It is very desirable the abscess should neither break into the rectum nor into the urethra, nor that the matter should insinuate itself behind the bladder, nor indeed go any where except to the surface. The same precautions should be observed, and the same practice followed, as in abscess by the side of the rectum, by making an early puncture. If matter should not follow on the first day, it generally will on the second, and the straight sharp-pointed bistoury should be used for this purpose, and pressed on, from the perineum through its deep fascia, by the side of the urethra, and above the rectum, until the surgeon is assured that it has penetrated the swelling; the flow of matter from which will prove the fact, and the slight bleeding which will ensue must under such circumstances do good. An abscess which is opened in this way, or which opens of itself in this manner, usually heals with little difficulty. If it opens into the rectum it is always a serious matter, and it is by no means readily cured when it bursts into the urethra. If it passes behind the bladder death is often the result, after a very prolonged illness, of which I have seen some very unhappy examples." 215.

Chronic abscess is more common than acute. It generally occurs between the ages of forty and sixty, and is usually but not always the consequence of stricture. It occasions or accompanies chronic inflammation of the mucous membrane of the bladder, and fatal disease of the kidney. Mr. Guthrie relates a striking instance of this complaint.

*Case.* An early friend of his was attacked about thirty years ago with uneasiness in the back part of the urethra, a great desire to make water and pain on passing it, without any discharge, but with a sense of fulness in the perineum, of weight in the hips and loins; the uneasiness was increased on evacuating the bowels. The gentleman was continually sitting with a lady to whom he was attached, but whom he could not marry. Three weeks of antiphlogistic treatment removed the symptoms in question. He experienced another attack at Lisbon in 1809; it yielded in a similar way. In the interval he had thrice had gonorrhœa, which each time was cured by copaiba. In 1817 he had another attack which was more obstinate, but ultimately yielded to a similar treatment, with the addition of a mild course of mercury. After this he married, and remained well for several years, but unhappily became a widower, and some months afterwards had a return of his complaint without any very evident cause. All the usual remedies now failed, and his disease gradually increased. During three years, he consulted all the most eminent surgeons in London, and at last died, completely exhausted, under the care of Dr. Prout. On opening the body, Mr. Guthrie found the prostate almost an empty sac, having been the seat of several abscesses communicating with the urethra. The internal surface of the bladder was in a state of chronic inflammation, but without ulceration, although, from the pain at the extremity of the penis, and the amazing quantity of discharge, almost apparently of a purulent nature mixed with the urine, considerable ulceration was expected. The ureters were much enlarged, and the kidneys diseased, that of the left side particularly being enlarged, and little more than an empty lobulated bag.

The treatment advised by Mr. Guthrie consists of injections into the bladder of warm water containing from half a grain to a grain of the acetate of morphia—the horizontal posture—and, after the matter has been discharged, change of air, tonics, the turpentines, and balsams, and opium. His remedies indeed are those of all judicious surgeons. Yet he has not alluded to a remedy as useful as any we have mentioned. It is the introduction and the maintenance of a seton in the perineum. We have found much benefit from its employment.

In every case of disease of the urethra, says Mr. Guthrie, great advantage and comfort will be derived from fomenting the perineum twice a day with a large sponge which has been dipped in hot water, or by sitting for a few minutes in the hip-bath.

Twenty-nine pages are occupied with the description of chronic enlargement of the prostate gland. Yet the subject admits of so little novelty that our notice on this head may be brief. We shall merely allude to three insulated circumstances.

1. The ancient opinion that chronic enlargement of the prostate gland partakes of the character of scirrhus, is rejected by the accurate pathological knowledge of the present day. For other organs and tissues in the body are not found to be altered in connexion with this complaint. Mr. Guthrie has, however, witnessed two cases in which the nearest absorbent glands appeared to be affected with chronic suppuration of a scrofulous description. In one individual, the prostate was larger than a closed hand, had partaken of a cheesy-like suppurative process of this kind, and the whole pelvis was nearly filled up by a mass of disease of a similar character.

2. When the prostate has become enlarged, the tumor prevents the complete evacuation of the bladder. The consequence of this is perhaps unexpected, and certainly remarkable. If, after the patient has attempted to empty the bladder of its contents, one or two ounces of urine are left in it, the desire to make water still continues, or scarcely subsides before it returns as strongly as ever, and requires a similar and unavailing effort to expel it. So that after a dozen trials half as many ounces only will be evacuated; whilst if the first two ounces be drawn off and the bladder is completely emptied, the patient will perhaps sleep for six or seven hours quietly; and when he awakes make the six or eight ounces of water at once, which he could only have done at a dozen trials without such precaution having been taken.

3. The last point is probably more speculative than practical. We shall suffer Mr. Guthrie to plead his own cause.

"A question has arisen in my mind, whether any operation could be done on the prostate from the perineum; and I was led to entertain it from finding that in a patient on whom I had operated for stone, whose prostate gland was much enlarged, I had rendered him a further service in the diminution of the prostate; so that instead of making his water with difficulty, he afterwards made it easily, and the catheter passed with facility, instead of meeting with a considerable obstacle at the neck of the bladder. In fact, I was satisfied I had cured or nearly so the disease of the left lobe of the prostate, which I found to be much enlarged during the operation. The success which ensued in this case, made me think the proceeding deserved further consideration, and I inquired of several of my surgical friends whether they had met with any thing of the kind. At that time none could speak affirmatively, with the exception of Sir William Blizard, who lent me an unpublished paper on the diseases of the prostate gland, which had been read at the Medical and Chirurgical Society in 1806, from which I have extracted the following observations and case." 252.

The observations and the case of the veteran surgeon may be properly reduced to this. That sometimes chronic abscess occasions or is attended with much surrounding thickening, and that free division or evacuation of the abscess will materially lessen if not remove the condensation. He applies this reasonable principle, or rather this indubitable fact to the instance of the prostate; and his case is one in which that gland was found after death to be greatly indurated and enlarged with a central follicle of pus in either lateral lobe. Sir William supposes that division might have been performed, and conjectures that it might have been successful. It is probable that the speculations of Mr. Guthrie and the case of Sir William Blizard will hardly be sufficient to tempt the surgeon to hazard a difficult and dangerous operation on a doubtful diagnosis for a dubious advantage.

Chronic thickening of the neck of the bladder, and acute and chronic inflammation of that organ occupy the fifteenth chapter of the work. Our attention may be usefully limited to the first.

The disease, says Mr. Guthrie, may commence at an early period of life, and though kept at bay by the employment of the catheter, is ready at all times and on any fit occasion to start into dangerous activity. It is generally, however, the disease of old age.

It usually begins in an insidious manner. The patient is ill, yet he knows not how; he has a more frequent desire to make water than formerly, particularly at night; it does not flow so readily nor so freely as it had usually done; and he is most free from irritation whilst his mind is particularly occupied, and especially after dinner, when he can often refrain from attempting to make water for four or five hours. The elasticity of the neck of the bladder is impaired, and the ordinary action of its muscular coat is incapable of perfectly emptying it. The urine does now flow so readily as heretofore, and the patient strains to expel it. He suffers uneasiness, which increases to pain, and this, though relieved on discharging a little urine, speedily returns; for the bladder is now never completely emptied, and the urine which remains is a source of great irritation, although the quantity is really inconsiderable. The urine at first is clear, afterwards it becomes a little turbid, small shreds of mucus or of fibrine are seen floating in it, soon to be followed by a deposit of the same character, which becomes in turn glairy, viscid, and even resembling pus. At first it is acid, and continues so until this deposit takes place in quantity, when the whole on standing may be alkaline, as in the enlargement of the prostate, although the newly-secreted urine is acid. In the commencement of the complaint the urine is not albuminous, but after a time it may become so.

The efforts to make water lead to the formation of pouches in the bladder, and to chronic, or acute inflammation of that organ. In short, the usual train of sequelæ is observed, which follow on obstruction to the free evacuation of the urine.

A glance at the preceding symptoms might convince the practical and unbiassed surgeon, that although Mr. Guthrie has faithfully described an affection hitherto unnoticed or neglected, those symptoms are not so determinate as might be wished. They are such as enlargement of the prostate may produce, and their character is probably too vague to allow a definitive diagnosis to be founded on them. Such at least appears to us to be the fact. Yet, perhaps, in opposition to this it may be urged that enlargement of the prostate, if present, may be ascertained. Be that as it may, we proceed to the next and the concluding chapter, in which Mr. Guthrie discusses the treatment of "the bar at the neck of the bladder." That treatment consists of suggestions rather than results. It may be closely packed.

When the disease, chronic thickening of the structure at the neck of the bladder occasioning what Mr. Guthrie terms "the bar," occurs in persons under or about the middle term of life, the steady use of the solid silver sound, gradually increasing the size of the instrument to the largest the urethra will permit, slowly accomplishes a cure. Yet the catheter, as in cases of stricture of the urethra, or enlargement of the prostate, must be passed now and then to prevent a relapse. In this, as in most diseases of the bladder, Mr. Guthrie is a zealous patron of the practice of washing out the bladder by injections of warm water, sometimes combined with opium or with morphia. Mr. G. has caught the spirit of Jesse Foot, whose ardent pamphlet on the manifold advantages of the vesicæ lotura may probably be familiar to some of our readers.

"The silver catheter or the solid sound are not however always competent to effect a cure, or even to give relief when passed from time to time. The incapability of emptying the bladder, of evacuating even an ounce of urine, remains unrelieved, although the silver catheter is introduced twice and even oftener in the twenty-four hours. The attempt to dilate the neck of the bladder by a larger or by a dilating instrument, only brings on pain and increases the evil, so that it is obliged to be given up for a smaller instrument than the one originally used, until the irritation thus caused shall have subsided. In these cases recourse must be had to the permanent catheter, or one which is retained constantly in the bladder, and which, by its slow and gradual operation, leads to the absorption of the bar or stricture in the same manner as it leads to the beating down of an enlarged portion of the prostate gland. I have reason to believe that I have succeeded in this way in many instances in affording relief, that could not be obtained by either of the other means indicated; but these cases may also have been accompaniad by disease of the prostate, a point which dissection alone could verify." 273.

But these means may fail, and the possibility or chance of advantage from an operation presents itself, of course, to the mind of the operating surgeon. Mr. Guthrie hints that "the bar" may be divided, and, growing more confident as he proceeds, he presently proposes to divide it. The instrument which he approves, and has twice employed, is a modification, perhaps an improvement of that of Mr. Stafford, containing a central perforator or lancet, capable of cutting on the side, and of being easily cleaned. When the nature of the case has been "well ascertained," the knife being projected just as the instrument is felt to be passing over the bar, will incise it, and if, after it has just passed into the bladder, it be withdrawn, the knife, in returning, will enlarge the incision. The latter, indeed, may be entirely made by the retiring motion of the instrument, the cutting blade of which is only unsheathed in its withdrawal. Mr. Guthrie allows that this is rather a suggestion, than the confident announcement of trial and success.

"If the bar be thin or narrow, I have no doubt of the possibility of dividing it in this way without doing mischief; and in two cases in which I have tried it, I have reason to believe the object was effected, from the greater facility with which the catheter afterwards passed into the bladder, and from the relief obtained in pasing the urine." 277.

Even in the latter periods of the case, when complications of a serious description have been the result of its continuance, Mr. Guthrie discovers a gleam of hope reflected from the blade of the scalpel.

"In the very advanced stages of the disease, when the bar is fully formed, when pouches exist, and the mucous membrane is in a state of chronic inflammation, giving rise to an almost continued and irresistible desire to make water, whilst the agony in passing a few drops is extreme; I believe it will be found, that an operation similar in its effects, but milder in its nature than that for the stone, is the one which ought to be had recourse to, if relief is to be obtained from operative surgery." 279.

We fear that few surgeons would venture, and few patients would permit, the performance of an operation resembling, though mildly, that for the stone. The suffering is certain and immediate, the danger not to be contemned, the advantage dubious and remote. Yet the candid reader will not hastily reject the specious proposition.

Here we must take our leave of Mr. Guthrie, a zealous, an active, and an able author. The length of our notice is sufficient evidence of the value we set on this gentleman's writings. A pert reviewer might select some passages for quibbling and for cavil; a generous critic regards the execution and the tenor of the whole—acknowledges the claims of observation applied to the elucidation of a complicated subject; and does not befoul with the harpy talons of a hidden and a hungry disputant, the table spread before him by one who stands honourably high with the profession.

